# Life-work balance of school-principals during the COVID-19 pandemic in Poland: Karina Leksy

**DOI:** 10.1093/eurpub/ckac129.658

**Published:** 2022-10-25

**Authors:** K Leksy, K Borzucka-Sitkiewicz, R Muster

**Affiliations:** University of Silesia, Katowice, Poland

## Abstract

**Background:**

The health crisis caused by the COVID-19 pandemic has severely affected the education sector and its whole community. Due to their responsibility for organizing schoolwork, school managers were in an extremely challenging position. The main objective of the present study is to reveal the extent to which school principals in Poland put aside their own needs in favor of fulfilling their professional duties during the COVID-19 pandemic.

**Methods:**

The results come from an online survey among school principals in Poland, which is part of the international COVID-19 Health Literacy School Principals Survey. The survey was conducted in 8 out of 16 provinces in Poland between June 2021 and December 2021. 1899 school principals participated in the survey, of which 928 completed the questionnaire.

**Results:**

The study revealed that 68,3% of school principals often and very often worked longer than contractually agreed and 71,3% reported to be available for their colleagues, pupils, and parents in their free time. Most of them also had to give up leisure activities in favor of work (67,9%), work extra hours in their spare time (60%), waive breaks during working hours (57,3%), and did not get sufficient sleep in favor of work (50,5%). Devoting more time to work and high stress levels during the pandemic were associated with somatic complaints among respondents (e.g. muscle pain (neck, shoulder, or back) and headache).

**Conclusions:**

The results suggest that Polish school principals worked at the expense of their free time and health during the COVID-19 pandemic. As such, findings emphasized a lack of life-work balance and the need to raise their awareness of the consequences of self-exploitation in work in challenging times. The ability to set healthy boundaries between work and private life among managers is one of the health promotion tasks in demanding times.

